# Allergic Diseases and Impaired Lung Function in Children Exposed and Unexposed to Artisanal Brick Production

**DOI:** 10.3390/children13060734

**Published:** 2026-05-25

**Authors:** Benigno Linares Segovia, Rocío Stephanie Bermúdez Pérez, Rebeca Monroy-Torres, Octavio Alejandro Jiménez Garza, Norma Amador Licona, Juan M. Guízar-Mendoza, Rodrigo Francisco del Río Hidalgo, Juan Antonio Ortega García, Luz Claudio

**Affiliations:** 1 Environmental Nutrition and Food Security Laboratory (LANAySA), Division of Health Sciences, Department of Medicine and Nutrition, University of Guanajuato, Blvd. Puente del Milenio 1001, Leon 37670, Guanajuato, Mexico; blinares70@gmail.com (B.L.S.); rs.bermudezperez@ugto.mx (R.S.B.P.); 2 Department of Pediatrics, Unidad Médica de Alta Especialidad, Salamanca 36730, Guanajuato, Mexico; 3 Psychology and Nursing School, Coahuila Autonomous University, North Unit, Carretera 57 Kilómetro 5, Monclova 25720, Coah, Mexico; ojimenezgarza@yahoo.com; 4 UMAE HE 1, Instituto Mexicano del Seguro Social, Blvd. Adolfo Lopez Mateos SN, Leon 37320, Guanajuato, Mexico; licoamador@gmail.com; 5 Departamento de Odontología, Campus Leon, Universidad De La Salle Bajío, Av. Universidad 602, Leon 37150, Guanajuato, Mexico; guizarjm@gmail.com; 6 Department of Pneumology, University of Guadalajara, Guadalajara 44340, Jalisco, Mexico; dr.delrio@hotmail.com; 7 Hospital Universitario de la Virgen Arrixaca, Universidad Murcia, 30120 Murcia, Spain; ortega@pehsu.org; 8 Division of International Health, Department of Environmental Medicine and Public Health, Mount Sinai School of Medicine, New York, NY 10029, USA; luz.claudio@mssm.edu

**Keywords:** brickyard, lung function, allergic diseases, children

## Abstract

**Highlights:**

**What are the main findings?**

- Evidence indicates that exposure to artisanal brick kilns increases respiratory symptoms, asthma risk, and pulmonary impairment, with children being particularly vulnerable.

**What does the study add to the literature?**

- Male sex and residence in brickyards were the principal risk factors for impaired lung function.
- Among children exposed to artisanal brick production, both overweight and underweight were more frequent than in unexposed peers.
- The frequency of respiratory symptoms (cough, wheezing, rhinorrhea, and acute respiratory tract infections) was higher in exposed children.

**What are the implications of the results obtained?**

- Nutritional findings such as overweight and obesity in boys and girls justify the inclusion of dietary and nutritional variables in these types of studies and designs to prevent adverse health effects, mainly in childhood. These results support integrating nutritional assessment and public policies to mitigate respiratory and nutritional risks in exposed children.

**Abstract:**

Background: During the firing stage of artisanal brick production, particulate matter and other pollutants are released into the air, to which children are particularly vulnerable. Objective: To compare the frequency of allergic diseases and impaired lung function among children exposed and unexposed to artisanal brick production. Methods: A cross-sectional comparative study was conducted among 386 children aged 6 to 14 years, recruited from a primary and a secondary school in Guanajuato, Mexico. Participants were classified as exposed (*n* = 193) or unexposed (*n* = 193) to artisanal brick production. Once parents and children voluntarily consented to participate, study procedures were initiated. Forced spirometry and anthropometric measurements were performed, and the International Study of Asthma and Allergies in Childhood (ISAAC) questionnaire was administered. We assessed the frequency of respiratory symptoms, allergic diseases, and lung function abnormalities. We measured the frequency of respiratory symptoms, allergic diseases, and lung function abnormalities. Results: The mean age of the 386 children was 9.7 ± 1.7 years. Each group consisted of 103 girls and 90 boys. The most important risk factors for impaired lung function were living in the brickyard (OR = 6.9, 95% CI: 4.3–11.1; *p* = 0.001) and being male (OR = 3.6, 95% CI: 2.3–5.7; *p* = 0.001). The prevalence of impaired lung function was 13.5% in exposed and 4.1% in unexposed children (OR = 3.6, 95% CI: 1.5–8.1; *p* = 0.001). Most of the respiratory symptoms observed were obstructive and we found no difference in the frequency of allergic diseases, but respiratory symptoms were more frequent in exposed children. Conclusions: Respiratory symptoms and obstructive abnormalities in pulmonary function are more prevalent among children living in brickyards. Male sex and residence in the brickyard were the principal risk factors for impaired lung function.

## 1. Introduction

Environmental air pollution is a major risk to human health and is estimated to cause approximately two million premature deaths worldwide each year [1]. The construction brick industry is recognized as a significant source of air pollution and is considered an environmental, social, and health problem globally. This activity is often conducted informally and is associated with the poorest communities, generating environmental and health damage through toxic smoke emissions, odors, and landscape alterations [2,3,4].

Traditionally, brick production has been a small-scale and unorganized industry, mainly concentrated in rural and peri-urban areas of developing countries. In these settings, brick manufacturing frequently involves entire families, with members engaged in kneading clay, preparing mixtures to form bricks, and transporting materials to be burned in kilns. Children are commonly involved in these tasks, which increases their vulnerability to harmful exposures [4,5]. The firing stage of brick production is the principal source of contamination, releasing large amounts of particulate matter, carbon monoxide, sulfur oxides, ozone, and hydrocarbons into the atmosphere [6]. People directly engaged in brick manufacturing are the most exposed; however, because most enterprises are family-based, women, children, and older adults are continuously subjected to these pollutants [3].

Epidemiological studies show that exposure to air pollution is associated with an increased incidence and severity of asthma [5,6], acute respiratory infections [7,8], pulmonary function decline [9] and other chronic obstructive pulmonary diseases [10,11].

Children have certain characteristics that make them more susceptible to environmental impact unlike adults [12]. Thus, from the point of view of the dose compared with adults, children breathe faster and play outdoors often; therefore, due to their lower weight, they are exposed to a greater dose per unit mass. Also, due to their physiologically immature immune system and organs, irritation and inflammation caused by contaminants can easily obstruct their airways [13]. In the state of Guanajuato, Mexico, artisanal brick production is a common activity. In Guanajuato, as in several other states, the growth of the real estate sector has perpetuated this activity under precarious conditions without progress towards regularization, especially in the city of León, which, in addition to being the most populated, has the largest number of brick kilns [14]. We hypothesized that children exposed to artisanal brick production would present a higher frequency of allergic diseases and impaired lung function compared to unexposed children. Therefore, the objective of this study was to compare these outcomes between exposed and unexposed groups, with the aim of informing and guiding health actions to mitigate the damage.

## 2. Materials and Methods

### 2.1. Study Population

We performed a cross-sectional comparative study, and we included children 6 to 14 years of age, exposed and unexposed to artisanal brick production. The required sample size was estimated at 136 participants to detect a 15% difference in the frequency of respiratory symptoms (cough) between those exposed and those not exposed to artisanal brick production, using the formula for comparing two proportions, with a one-sided alpha of 0.05, a beta of 0.20 and a power of 0.80. The expected 15% difference between schools was obtained from a previously reported frequency in children exposed to contaminants in a city in Guanajuato [9].

This study took place from September 2011 to March 2012, in the “Ladrilleras del Refugio” community (exposed), which has approximately 300 brick kilns; a total of 80 to 90% of the population is engaged in artisanal brick production. The community is located on the outskirts of the city of Leon, Guanajuato, Mexico. Leon is the most populated city from Guanajuato State; it has a territorial extension of 1200 km^2^ (3.9% of the total state surface), 1.5 million inhabitants and a population density of 1250 inhabitants per km^2^. Its climate is temperate most of the time, and it forms part of the industrial corridor of the state. The community of Ladrilleras del Refugio in Leon, Guanajuato, contains the largest number of brick kilns in the municipality, causing highly polluting emissions into the atmosphere of the city and its surroundings. Among the main pollutants emitted by the brick kilns are particulate matter (PM), carbon monoxide (CO), nitrogen dioxide (NO_2_), sulfur dioxide (SO_2_), metals, and black carbon (BC), resulting from incomplete combustion [5].

The participants were paired with children from 6 to 14 years of age from a colony of Silao, Guanajuato (unexposed), a municipality with agricultural and industrial activity, located 25 km from the state capital and 15 km from the municipality of León. Its territorial extension is 531.42 km^2^ (1.7% of the total area of the state) and it has 189,567 inhabitants. The climate is predominantly temperate, and the municipality forms part of the state’s industrial corridor [15]. According to reports from the Guanajuato State Ecology Institute, the municipality of Silao has the lowest concentration of pollutants compared to other municipalities in the industrial area.

Children were selected in both schools (cases and controls) through simple random sampling from the school’s nominal census, that is, from the list of students enrolled in each group. The selection criteria were, being a boy or girl, with a minimum age of 6 and a maximum age of 14 years and being originally from the study area with at least 3 years of residence. Since spirometry testing was required, children were not included if they had heart disease, cleft lip, or cleft palate. The selection process, as well as the measurements, were carried out by the research team, which consisted of a nurse, a pediatrician, and a nutritionist. For the selection of the control school, a multistage probabilistic sampling design was employed: once the basic geo-statistical area corresponding to the city of Silao was identified, a primary school was chosen, and the selection of children was then performed, as previously described and in the same manner as for the cases, using simple random sampling.

### 2.2. Study Design

A detailed clinical history was taken and physical examination performed on each child. The medical and sociodemographic history were obtained through the questionnaire proposed by the International Study of Asthma and Allergies in Childhood (ISAAC) validated in a previous study [9]. Permission was obtained from the State Department of Education and the participating schools. Parents and each participant were informed about the study’s objective and procedures before providing informed consent. Clinical and demographic information was obtained using the questionnaire developed by the International Study of Asthma and Allergies in Childhood (ISAAC), validated for Spanish [9]. Exposure to tobacco smoke in participants and their parents was investigated, as well as the use of commonly used indoor fossil fuels (including charcoal and firewood) in Mexico. Children ≤ 9 years of age and their parents answered together, while older children answered individually. Questions were asked about respiratory symptoms (wheezing, rhinorrhea, respiratory infection, dyspnea, and hospitalization secondary to respiratory infection). Participants and their families were asked about the history of atopy. Socioeconomic level was estimated using family income, considering the educational level of the parents and the number of rooms in the dwelling. The study was approved by the Research Committee of the Faculty of Medicine (now called: Department of Medicine and Nutrition) of the University of Guanajuato with registration number 360-10.

Child weight and height were measured and age- and sex-specific z-scores for body mass index (zBMI) were calculated using the WHO growth reference 2007. Thinness, overweight and obesity were defined using the same reference. All measurements were performed by the dietician, using the methodology of the International Society for the Advancement of Kineanthropometry (ISAK). Weight was measured with a portable scale (Seca^®^ 813, Hamburg, Germany) with a 125 kg maximum capacity and a ±100 g error. Height was measured by stadiometer with a precision of 0.1 cm (Seca^®^ 222, Hamburg, Germany).

It was considered an allergen when a positive response to the following items were considered as allergens: carpets at home (mites), corresponding to item 10; living with animals such as dogs, cats, pigeons, and birds (items 11 to 15); and the presence of cockroaches at home (item 16). Atopy was considered as a maternal or paternal history of atopy, as well as a diagnosis of atopy in the patient established by a physician or pediatric specialist. For the diagnosis of asthma, the following key questions were used regarding the past 12 months, as they are the most sensitive to persistent asthma. Wheezing, have you been wheezing in your chest in the last year? (This is the most sensitive indicator). Frequency, how many wheezing attacks have you had? Sleep: Have you been awakened at night by wheezing? Exercise, have you experienced wheezing after physical activity? Asthma was diagnosed when the child had episodic cough, breathlessness, and wheeze responsive to bronchodilators with or without steroids. Lung function was measured by forced spirometry. The spirometry was performed with a spirometer EasyOne^®^ (NDD, Medizintechnik AG, Zurich, Technopark darned Switzerland), which meets the diagnostic criteria for precision, accuracy and linearity, established by the American Thoracic Society [16]. To carry out the recommendations of the ATS and the following parameters were obtained: Forced Vital Capacity (FVC), Forced Expiratory Volume in one second (FEV1) and FVC/FEV1 ratio. The quality of spirometry tests was assessed by several criteria in addition to the automatic evaluation done by the software device. One was the number of acceptable maneuvers according to ATS, 1 ranging from 0 to 3, the highest kept by the spirometry software. Another indicator of quality was reproducibility where FEV1 and FVC were considered reproducible according to ATS criteria when the best two trials differed by not more than 200 mL. A total of 97.5% of the tests achieved reproducibility within 150 mL fulfilling the 2005 ATS-ERS criteria [17]. Reference values [18] for Mexican-Americans were used, considering that children > 7 years old can fulfill ATS criteria of quality after the first spirometry evaluation. The presence of spirometry values < 5% of the reference value according to height, weight and age will be considered abnormal. The obstructive pattern was define by the diminution of the FEV1 and quotient FEV1%/FVC; and the non-obstructive pattern by diminution of the FVC, without change or with increase (>95%) of quotient FEV1%/FVC; and the mixed pattern in case of diminution of FEV1 and of FVC. Spirometry tests that met acceptability and repeatability criteria were included; only spirometry tests with quality grade A, B, and C were included, with variability of less than 150 mL or less than 5% between the best and second-best values for FVC and FEV1. The spirometry tests were performed and verified by a NIOSH/OSHA-certified spirometry technician (National Institute for Occupational Safety and Health—NIOSH and Occupational Safety and Health Administration—OSHA).

### 2.3. Statistical Analysis

All data were expressed as mean ± SD or as median and 95% CI according to their normal distribution. Comparison of demographic and clinical data was performed using *X*^2^ or Student’s *t* test according to the type of variable. Stepwise multiple regression was performed with impaired lung function as the dependent variable and group, age, gender, body mass index, the antecedent atopy, exposure to allergens and passive smoking as regressors. For statistical analysis, we used Stata 9.0 (Stata Corporation, College Station, TX, USA) and SPSS 21.0 Statistical Software.

## 3. Results

### 3.1. Clinical Characteristics

We studied 386 children (193 exposed and 193 unexposed), with a mean age of 9.7 ± 1.7 years, where each group had 103 girls and 90 boys. Both parents and children invited to participate provided informed consent and assent. Exclusions occurred during the study when spirometry tests did not meet the quality control standards established by the American Thoracic Society, representing an average of 2% of the sample in both the case and control groups. The economic income per family was significantly lower in the families of the children exposed to brick artisan production (4.0 vs. 2.7 dollars on average per day, *p* = 0.001), but both groups were classified at a low socioeconomic level. All participants had health coverage, although with differences in the institution. In the area of brickwork, the Popular Insurance (Seguro Popular, a public health program that provided free or low-cost medical coverage to uninsured populations in Mexico and was in effect until 2020) prevailed (33.8%), whereas in the unexposed group the Mexican Institute of Social Security predominated (42.5%). The weight, height and body mass index were significantly lower in children exposed to artisanal brick production (Table 1). Fifty-nine percent of the subjects were classified as eutrophic, the rest had some alteration of nutritional status; malnutrition and underweight predominated in the exposed, 10.9% vs. 2.6% (OR = 4.2, 95% CI: 1.6–10.9), while overweight and obesity were more common in the unexposed, 50.1% vs. 16.1% (OR = 4.2, 95% CI: 1.6–10.9).

### 3.2. Allergic Diseases, Respiratory Symptoms and Pulmonary Function Measures

Based on the ISAAC questionnaire, the frequency of respiratory symptoms (cough, wheezing and acute respiratory tract infection) was more frequent in children exposed. There was no significant difference in the frequency of allergic diseases (Table 2).

Multiple regression analysis showed that the factor most strongly associated with wheezing was living in a brickyard area (OR = 1.98, 95% CI: 1.01–3.8); rhinorrhea was associated with a history of atopy (OR = 1.37, 95% CI: 1.01–2.6). The factors most strongly associated with asthma were being male (OR = 7.4, 95% CI: 1.3–40.7) and exposure to allergens (OR = 6.1, 95% CI: 1.2–30). On the other hand, the factor most strongly associated with rhinitis was living in a brickyard area (OR = 3.3, 95% CI: 1.9–5.7). No history of atopy was a protective factor for eczema (OR = 0.26, 95% CI: 0.10–0.63). The factor most associated with hospitalization for respiratory infections was being male (OR = 2.0, 95% CI: 1.09–3.7).

Compared with unexposed children, all lung function parameters were significantly lower in children living in “Ladrilleras del Refugio”. According to spirometry values, impaired lung function was higher in exposed children (OR = 3.6, 95%CI: 1.5–8.1). Also, obstructive lung function impairment was more common in the exposed group; no significant differences were observed in non-obstructive and mixed disorders (Table 2). Logistic regression analysis showed that residing in the “Ladrilleras del Refugio” community and being male were the most important risk factors for impairment lung function (Table 3).

## 4. Discussion

In our series, living in the community of “Ladrilleras del Refugio” and being male were the most important risk factors for deterioration in lung function, with 3.6 (OR) greater odds (CI = 2.3–5.7) compared to girls. Children in this community are also at increased risk of malnutrition, both overweight and underweight for age, reflecting the precarious living conditions of families engaged in artisanal brick production. Poverty places them at a disadvantage, hindering their integration and development, and previous reports indicate high levels of anemia, malnutrition, frequent illness, and low IQs among Mexican children in similar settings [5,19].

Children are physiologically more susceptible to environmental threats due to their reduced ability to metabolize toxic substances, and this vulnerability is compounded by poverty, malnutrition, and unfavorable environments [13,20]. These conditions explain the higher frequency of respiratory symptoms and impaired lung function observed in exposed children compared with unexposed peers; these results are consistent with the study by Kashyap et al. [21], where the most exposed group showed significantly higher air pollution levels and a higher prevalence of obstructive and restrictive patterns in pulmonary function compared to the unexposed group. As in other parts of the world, artisanal brick production is a family business, often involving children, and in Mexico child labor is closely linked to domestic economy and family organization patterns [22].

Artisanal brick production represents a significant source of pollution, yet remains unregulated, and its health and environmental costs are underestimated by policymakers [23]. Fuels used in kilns include wood, sawdust, fuel oil, diesel, domestic garbage, and even tires, releasing complex mixtures of toxic compounds such as CO, NO_x_, SO_x_, VOCs, dioxins, PM10, and polycyclic aromatic hydrocarbons (PAHs) [24,25]. Exposure to these pollutants has been linked to respiratory impairment and may contribute to more than 10% of the cancer burden worldwide [1].

Our findings show that children exposed to artisanal brick production present a significant decrease in pulmonary function parameters, particularly FEV1 and the FEV1/FVC ratio, with a predominance of an obstructive pattern. This result coincides with studies in India, Bangladesh, Chile, and Brazil, where exposure to biomass and fossil fuel pollution has been associated with reduced FEV1 and increased risk of asthma in children [1,2,5,6]. The biological mechanism involves chronic inhalation of particles and gases that induce bronchial inflammation, airway hyperreactivity, and epithelial remodeling, leading to progressive obstruction. In children, the smaller airway diameter and immature immune system exacerbate these effects, resulting in greater susceptibility to recurrent infections [26,27,28].

Limitations of this study include the time gap between data collection (2011–2012) and publication, which may restrict immediate applicability, and the cross-sectional design, which prevents establishing causality. Additionally, environmental exposure was inferred by residence rather than direct pollutant measurements, and symptom reporting may be subject to recall bias. Socioeconomic factors such as low income, malnutrition, and precarious housing must also be considered as confounders, as they amplify vulnerability and reduce access to healthcare [7,28].

Future research should include longitudinal studies to assess progression of lung function into adolescence and adulthood, direct measurements of pollutants to strengthen exposure–response associations, and integration of nutritional and inflammatory biomarkers to better understand the interaction between malnutrition, pollution, and respiratory health. Community interventions promoting cleaner combustion technologies and health education programs could provide evidence on effective strategies to mitigate harm. Finally, incorporating climate-related dimensions, such as the impact of fuel combustion on greenhouse gas emissions, would broaden the perspective on how artisanal brick production affects both respiratory and nutritional health and contributes to climate change, opening opportunities for integrated health, environmental, and sustainability interventions [5].

## 5. Conclusions

Compared with unexposed, children living in a brickyard have a higher frequency of alterations in lung function, predominantly obstructive pattern and mixed. The frequency of respiratory symptoms (cough, wheezing, rhinorrhea and acute respiratory tract infection) was more frequent in children exposed. There was no significant difference in the frequency of allergic diseases. In our series, being male and living in a brickyard represented a higher risk for impaired lung function. In children exposed to artisanal brick production, overweight and low weight for age is greater than in unexposed.

## 6. Health Recommendations

1. Implement community-based programs aimed at reducing emissions from artisanal brick kilns, promoting cleaner technologies and providing training for producers to minimize children’s exposure to airborne pollutants.
2. Strengthen epidemiological surveillance and school-based respiratory health campaigns, including periodic lung function screenings for children living near brickyards and educational initiatives for families on risks and protective measures.

## Figures and Tables

**Table 1:** Clinical characteristics of the study populations.

	Artisanal Brick Production		
	Exposed *n* = 193	Unexposed *n* = 193	*p* Value
Male/Female	90/103	90/103	0.99
Age (years)	10 (9–10)	10 (9–10)	0.76
Weight (kg)	29.8 (27.4–32.7)	39.8 (38.7–41.0)	0.0001
Height (m)	1.34 (1.31–1.37)	1.42 (1.41–1.43)	0.0001
BMI (kg/m^2^)	16.7 (16.2–17.1)	19.1 (18.7–19.8	0.0001
Female	16.6 (16.0–17.3)	18.9 (18.3–19.8)	0.0001
Male	16.7 (16.0–20.1)	19.6 (18.8–20.6)	0.0001
FVC (liters)	2.0 ± 0.5	2.6 ± 0.7	0.0001
FVC (% predicted)	100.0 ± 17.5	109.52 ± 16.0	0.0001
FEV1 (liters)	1.7 ± 0.4	2.1 ± 0.5	0.0001
FEV1 (% predicted)	95.2 ± 15.4	103.4 ± 13.8	0.0001
FEV1/FVC%	83.2 ± 7.6	89.1 ± 1.3	0.0001
PEFR (L/min)	4.1 ±1.0	5.0 ± 1.1	0.0001
Clinical history *n* (%)			
Atopy	135 (70.1)	17 (8.8)	0.03
Allergens	154 (80.4)	29 (15.0)	0.0001
Fossil fuels	118 (61.3)	59 (30.3)	0.0001
Passive smoking	85 (43.8)	53 (27.7)	0.0001
Economic income (USD/week)	39.2 USD (31.0–44.8)	168.1 USD (140.1–179.3)	0.0001

Notes: BMI: Body Mass Index, FVC: Forced Vital Capacity, FEV1: Forced Expiratory Volume in One Second, PEFR: Peak Expiratory Flow Rate, USD: Dollars per week.

**Table 2:** Prevalence of respiratory symptoms, allergic diseases and impaired lung function.

Artisanal Brick Production				
	Exposed *n* = 193 No. (%)	Unexposed *n* = 193 No. (%)	OR (95% CI)	*p*
Respiratory symptoms				
Cough	28 (14.5)	14 (7.3)	2.1 (1.1–4.2)	0.02
Wheezing	25 (13.0)	12 (6.2)	1.5 (1.1–2.5)	0.02
Rhinorrhea	50 (25.9)	47 (24.2)	1.0 (0.8–1.3)	0.72
ARTI ^&^	19 (9.8)	4 (2.1)	4.7 (1.6–13.7)	0.001
Allergic diseases				
Asthma	7 (3.6)	10 (5.2)	0.8 (0.55–1.2)	0.58
Rhinitis	4 (2.1)	2 (1.0)	1.5 (0.4–1.3)	0.41
Eczema	13 (6.7)	8 (4.1)	1.6 (0.6–4.1)	0.26
Impaired lung function	26 (13.5)	8 (4.1)	3.6 (1.5–8.1)	0.001
Obstructive	25 (12.9)	6 (3.1)	4.6 (1.8–11.5)	0.001
No Obstructive	1 (0.61)	2 (1.0)	0.5 (0.04–6.1)	0.63
Mixed pattern	0	0	-	-

ARTI ^&^: Hospitalization for acute respiratory tract infections.

**Table 3:** Factors associated with impaired lung function.

Variable	Beta	OR ^†^	95% CI
Male	1.30628	3.6 *	2.3–5.7
Allergen	0.0205	1.0	0.5–1.9
Atopy	−0.37398	0.6	0.3–1.3
Living in brickyard	1.93515	6.9 *	4.3–11.1
Malnutrition	−0.15476	0.8	0.5–1.3
Fossil fuels	0.29477	1.3	0.8–2.0
Passive smoking	0.06272	1.0	0.6–1.6

^†^ Odds ratio (95% CI) in multilevel logistic models with slope as random effects, adjusted by height, BMI, sex and age; clustering by child. * *p*-value < 0.05.

## Data Availability

The datasets generated during and/or analyzed during the current study are available from the corresponding author on reasonable request.

## Abbreviations

FEV1	Forced Expiratory Volume in One Second
BMI	Body Mass Index
ISAAC	International Study of Asthma and Allergies in Childhood
ATS	American Thoracic Society
FVC	Forced Vital Capacity
ERS	European Respiratory Society
SD	Standar Desviation
CI	Confidence Interval
CO	Carbon monoxide
NO_x_	Nitrogen oxides
SO_x_	Sulphur oxides
VOCs	Volatile organic compounds
PM	Breathable particulate matter
PM10	Breathable particulate matter with diameters of 10 microns
PAHs	Polycyclic aromatic hydrocarbons
IQ	Intelligence coefficient
